# 7.1 ELECTRORETINOGRAPHIC ANOMALIES IN SCHIZOPHRENIA AND THEIR RELATIONSHIPS WITH RETINAL STRUCTURE, VISUAL FUNCTIONS, CLINICAL SYMPTOMS, AND MEDICAL COMORBIDITIES

**DOI:** 10.1093/schbul/sby014.023

**Published:** 2018-04-01

**Authors:** Steven Silverstein, Docia Demmin, Molly Erickson, Judy Thompson, Danielle Paterno, Roni Netser

**Affiliations:** Rutgers University

## Abstract

**Background:**

Although several studies have documented retinal cell dysfunction in schizophrenia (Silverstein & Rosen, Scz Res: Cogn, 2015), the extent to which these abnormalities contribute to, and/or result from, other features of the condition is unclear. Thus we sought to: 1) evaluate associations between retinal signaling anomalies as measured with flash electroretinography (fERG) and previously reported changes in visual evoked potentials (VEPs), contrast sensitivity, visual acuity, and contour integration in people with schizophrenia (Silverstein, Neb Symp Motiv, 2016); 2) determine whether fERG anomalies are related to retinal structural abnormalities as indicated by optical coherence tomography (OCT); 3) examine relationships between fERG changes and psychiatric symptoms; 4) determine relationships between fERG anomalies and frequent medical comorbidities in schizophrenia that are known to affect the retina (e.g., diabetes, hypertension); and 5) examine potential medication effects on these findings.

**Methods:**

We have assessed 25 patients with schizophrenia and 25 controls who are free of medical comorbidity with fERG and measures of visual function and symptom severity, and data collection is ongoing with patients and controls with diabetes and/or hypertension using these same measures. In addition, we are in the process of completing data collection with two additional groups of patients and controls, one with fERG and OCT (n=12 to date), and another with fERG and VEPs (n=13 to date). fERG data are being collected under both light- and dark-adapted conditions, using a range of flash intensities, backgrounds, and temporal frequencies. The primary fERG variables of interest are a-wave and b-wave amplitudes, which reflect photoreceptor and bipolar cell responses, respectively, and the photopic negative response (PhNR), which reflects ganglion cell activity.

**Results:**

On photopic fERG tests, patients with schizophrenia demonstrated significantly weaker photoreceptor response when a flash was presented against an unlit background (p<.05), and during a steady-state flicker test (p<.005). On scotopic tests, the rate of response gain per unit of intensity increase was significantly weaker for patients than controls (p=.001). In both light- and dark-adapted conditions, patients demonstrated weaker signaling of bipolar cells (ps < .005). The schizophrenia group was also characterized by a weaker PhNR (p<.05). Weaker retinal cell responses were related to contrast sensitivity impairments in the schizophrenia group (ps < .05 and .001), but not to visual acuity or contour integration. Reduced responsiveness to low-intensity light was related to more severe negative symptoms, suggesting a reduced dynamic range within which environmental events (i.e., salience) are represented. Measures of retinal cell function were not related to antipsychotic medication dose. Preliminary findings indicate that attenuated fERG signals are not associated with weaker visual cortical responses (EEG-measured VEPs), presumably due to gain control mechanisms. We will report on the extent to which fERG anomalies are related to retinal structural changes and comorbid medical conditions.

**Discussion:**

Reduced signaling of photoreceptor, bipolar, and ganglion cells are characteristics of schizophrenia, and are not related to extent of antipsychotic medication use. These changes are related to reduced contrast sensitivity and increased negative symptoms, and may reflect an attenuated ability to accurately represent changes in the intensity of environmental stimuli. Data collection is ongoing for studies examining relationships between ERG indices and VEPs and medical comorbidities.

